# Proteases: Role and Function in Cancer

**DOI:** 10.3390/ijms23094632

**Published:** 2022-04-22

**Authors:** Janko Kos

**Affiliations:** Department of Biotechnology, Faculty of Pharmacy, Jožef Stefan Institute, University of Ljubljana, 1000 Ljubljana, Slovenia; janko.kos@ffa.uni-lj.si

The Special Issue “Proteases: Role and Function in Cancer” aimed to focus on basic and translational research to highlight the role of peptidases in tumor development and to assess their potential in cancer diagnosis and therapy. Peptidases are involved in various stages of cancer progression. The origin of harmful proteolytic activity can be tumor cells themselves. However, a growing number of studies have shown that peptidases associated with other cells in tumor microenvironment contribute significantly in pathological processes. Increased peptidase expression and/or activity create conditions favorable for tumor invasion, angiogenesis, and metastasis. Moreover, peptidases are important in modulating apoptosis and antitumor immune response as well as in the growth and development of cancer stem cells and the transition between epithelial and mesenchymal cell types.

In this issue, the papers are focused on two peptidase groups: metallo peptidases and cysteine peptidases. The role of metallo peptidases in tumor progression is well documented. They act predominantly extracellularly and affect tissue integrity, immune cell recruitment, and tissue turnover by degrading extracellular matrix (ECM) components and by releasing matrikines, cell surface-bound cytokines, growth factors, or their receptors. In a review paper, Niland et al. [1] examined the current knowledge on the role of various matrix metallopeptidases (MMPs) in cancer progression in context on subcellular and cellular level with a focus on MMP14. Besides the contribution of individual peptidases, the collective role and the possible coordination between MMP members seem to be important. Buttacavoli et al. [2] performed a multi-omics analysis of MMPs expression and the impact in genetic and epigenetic alterations in colorectal cancer using data mining and experimental investigations. The results underlined MMPs as cancer promoting factors (but in some cases also as suppressors). They showed that MMP2 and MMP9 expression correlates with the immune markers and that the interaction network of their co-expressed genes is connected with epithelial to mesenchymal transition (EMT) and immune response. MMP-2 and MMP-9 have also been considered as possible biomarkers in breast cancer to predict prognosis and metastasis development [3]. However, targeting MMPs is a complex task given that the action of individual MMPs may differ between cancer type, distinct stages of cancer progression, and even between individuals. As shown by Camacho et al. [4] in a cohort of 154 patients with breast cancer, a role of MMP-2 and MMP-9 as biomarkers for the prediction of progression and metastasis was not supported However, MMP-1 and MMP-3 appeared in this study as potential diagnostic biomarkers.

Cook et al. [5] present a role of the metalloprotease-disintegrin ADAM8 in the progression of pancreatic cancer. ADAM8 was suggested to be involved in cell–cell communication as its expression has been observed in tumor and immune cells. The authors analyzed extracellular vesicle release from pancreatic ductal adenocarcinoma cells and the cellular interactions with macrophages. In extracellular vesicles, ADAM8 levels positively correlated with those of MMP9 and lipocalin 2. They showed that the regulation of MMP-9 is independent of the M1/M2 polarization state, whereas lipocalin 2 expression is preferentially affected by M1-like macrophages. ADAM8 therefore has a systemic effect in the tumor microenvironment, and it would be worth involving in further studies other immune cell types such as neutrophils and natural killer cells in which ADAM8 is also highly expressed.

In contrast to metallo peptidases the function of cysteine peptidases can be either intracellular or extracellular. Inside the cells, they either act in lysosomal/endosomal vesicles either as a part of general intracellular catabolism or are involved in various regulatory functions. Two papers in this Special Issue are focused on the role of cysteine carboxypeptidase cathepsin X in cancer. Cathepsin X overexpression was demonstrated in several cancers wherein it promotes tumor progression by conferring resistance to apoptosis, enabling EMT, by enhancing invasion through interaction with integrin receptors ανβ3 and ανβ5, and by cleavage of tumor suppressor protein profilin-1 [6]. Majc et al. [7] demonstrated upregulated cathepsin X expression and activity in human glioblastoma multiforme (GBM) tissues compared to low-grade gliomas and nontumor brain tissues. Cathepsin X was localized in GBM cells as well as in tumor-associated macrophages and microglia. Selective cathepsin X inhibitors decreased the viability of patient-derived GBM cells as well as macrophages and microglia. They also examined the expression pattern of neuron-specific enzyme γ-enolase, which is also a target for cathepsin X, and found a correlation between high proteolytic activity of cathepsin X and *C*-terminal cleavage of γ-enolase. A strong co-localization between cathepsin X and γ-enolase, found in GBM tissues (preferentially in GBM-associated macrophages and microglia) confirms the involvement of cathepsin X in GBM progression and designates it as a potential target for therapeutic approaches.

The role of cathepsin X is important also in antitumor immune response. Previous studies indicated that the migration and adhesion of T cells and dendritic cells are affected by diminished cathepsin X activity. Leukocyte specific β2 integrin receptors such as LFA-1 can be sequentially trimmed at C-terminus by cathepsin X, which modulates their association with adaptor proteins and fine-tunes their affinity for extracellular ligands. It was shown that the overexpression of cathepsin X in T cells significantly increased their migration through LFA-1 receptor [8]. Jakoš et al. [9] tested whether cathepsin X activity regulates also immunological synapse stability of CD4+ T cells, which also depends on LFA-1. They did not confirm the involvement of cathepsin X in LFA-1 mediated regulation of immune synapse. However, by investigating cellular compartmentalization of cathepsin X they demonstrated that in NK cells cathepsin X preferentially colocalizes with perforin during cytotoxic granule release and is secreted during the process of degranulation. Cathepsin X inhibition, potentially used in anticancer therapy, would therefore not be detrimental to the NK cell cytolytic activity and would not impair immune synapse formation.

The activity of cysteine cathepsins is tightly regulated by their endogenous inhibitors (i.e., cystatins). They are in general nonspecific to particular cathepsins and are present predominantly in cell cytoplasm and in body fluids [10]. Cystatin F is an exception as it is capable of entering endosomal/lysosomal vesicles in which it directly regulates the activity of intracellular cysteine cathepsins [11]. It is predominantly expressed in immune cells and shown as an important regulator of the granzyme/perforin mediated cytotoxicity of natural killer (NK) and CD8+ T cells, the most important cytotoxic effector cells in antitumor immune response. Perišič Nanut et al. [12] also demonstrated that CD4+ T cells may acquire cytotoxic functions. They examined CD4+ human T cell clones derived from activated peripheral blood lymphocytes for the expression of cytotoxic machinery components and found that they express granzyme activators cysteine cathepsins C and H (as well as their inhibitor cystatin F). They propose cystatin F as a regulator of CD4+ T cell dependent antitumor immune response. 

Cathepsins can also reside in the cytoplasm and are able to cleave and modulate a number of biochemically significant signaling pathways; among them is an apoptosis. Regarding the latter, they can regulate the intrinsic and extrinsic signaling cascades, and exhibit both cancer pro- and anti-apoptotic activity. B-cell lymphoma (Bcl-2) family proteins have been well documented as substrates for cathepsins [13]. Soond et al. [14] outlined the rationale for therapeutically targeting of cathepsins in the context of either selectively abrogating their activity for the breakdown of the pro-apoptotic Bcl-2 proteins, or to maintain their activity for the breakdown of the anti-apoptotic Bcl-2 proteins. Their main goal is to favor apoptosis of cancer cells and, accordingly, to arrest tumor progression. The authors also present the therapeutic design of BH3-mimetics to achieve these goals. Apoptotic evasion is a hallmark of cancer. Besides the above-mentioned cathepsins and Bcl-2 proteins, caspases, a family of cysteine-aspartic peptidases, have a critical role in the activation and initiation of apoptosis. As with several other proteins, the role of caspases in cancer is also complex, with a potential to prevent or to promote tumorigenesis. Hounsell and Fan [15] reviewed the major findings in *Drosophila* on the dual role of caspases in tumorigenesis. As the activation of apoptosis is the end goal in cancer treatments, including chemotherapy and radiotherapy, a comprehensive understanding of the caspase function may improve the existing anti-cancer therapeutic approaches. 

To summarize, peptidases are key effector molecules involved in tumor progression. However, their function is complex and depends on peptidase catalytic mechanism, substrate specificity, localization, regulation, cancer cell type, and cancer stage, and there is no general approach how to impair their tumor associated proteolytic activity. In this Special Issue, several specific roles of peptidases are presented revealing either cancer promoting or suppressing functions. Only detailed understanding of pathological mechanisms enables a design of new therapeutic tools directed to harmful proteolytic activity in tumors, not affecting at the same time proteolytic fractions involved in antitumor responses or other physiological processes. 

## Data Availability

Not applicable.
